# Locally Performed HRD Testing for Ovarian Cancer? Yes, We Can!

**DOI:** 10.3390/cancers15010043

**Published:** 2022-12-21

**Authors:** Gilda Magliacane, Emanuela Brunetto, Silvia Calzavara, Alice Bergamini, Giovanni Battista Pipitone, Giovanna Marra, Miriam Redegalli, Greta Grassini, Emanuela Rabaiotti, Gianluca Taccagni, Lorenza Pecciarini, Paola Carrera, Giorgia Mangili, Claudio Doglioni, Maria Giulia Cangi

**Affiliations:** 1 Pathology Unit, IRCCS San Raffaele Scientific Institute, 20132 Milan, Italy; 2 Laboratory of Clinical Molecular Biology, IRCCS San Raffaele Scientific Institute, 20132 Milan, Italy; 3 Obstetrics and Gynecology Unit, IRCCS San Raffaele Scientific Institute, 20132 Milan, Italy

**Keywords:** BRCA, HRD, HRR, NGS, molecular testing, ovarian cancer, PARPi

## Abstract

**Simple Summary:**

Poly (ADP-ribose) polymerase inhibitors (PARPis) have been recently approved by international medicine agencies for the treatment of ovarian cancer patients with either BRCA pathogenic variants or homologous recombination deficiency (HRD), changing the ovarian cancer treatment landscape in both first-line and relapsed disease settings. Thus, assessing HRD is now crucial for patient management, making it a pressing need to offer an alternative to expensive and time-consuming outsourced analysis. The aim of the present study was to demonstrate the feasibility of locally performed HRD testing, using an easy and affordable CE-IVD NGS panel that enabled the analysis of HRD status within 5 days, with perfect concordance with Myriad results. Importantly, this strategy could also be applied in the near future to stratify patients with different tumor types, including breast cancer, pancreatic cancer, and prostatic cancer, thereby expanding the number of patients who could benefit from PARPi treatment.

**Abstract:**

Assessment of HRD status is now essential for ovarian cancer patient management. A relevant percentage of high-grade serous carcinoma (HGSC) is characterized by HRD, which is caused by genetic alterations in the homologous recombination repair (HRR) pathway. Recent trials have shown that not only patients with pathogenic/likely pathogenic *BRCA* variants, but also *BRCAwt*/HRD patients, are sensitive to PARPis and platinum therapy. The most common HRD test is Myriad MyChoice CDx, but there is a pressing need to offer an alternative to outsourcing analysis, which typically requires high costs and lengthy turnaround times. In order to set up a complete in-house workflow for HRD testing, we analyzed a small cohort of HGSC patients using the CE-IVD AmoyDx HRD Focus Panel and compared our results with Myriad’s. In addition, to further deepen the mechanisms behind HRD, we analyzed the study cohort by using both a custom NGS panel that analyzed 21 HRR-related genes and FISH analysis to determine the copy numbers of *PTEN* and *EMSY*. We found complete concordance in HRD status detected by the Amoy and the Myriad assays, supporting the feasibility of internal HRD testing.

## 1. Introduction

Ovarian cancer is the second most prevalent type of cancer in women over the age of 40 in developed countries, and it is the fifth leading cause of cancer-related death in women [1,2]. Importantly, it is the most lethal gynecologic cancer, having a 5-year survival rate of only 49.7% [3], and because it lacks distinct symptoms and specific biomarkers for early detection, it is frequently diagnosed at an advanced stage, after spreading beyond the pelvis [4,5]. High-grade serous carcinoma (HGSC) represents the most common type of ovarian cancer. HGSC is a genetically unstable tumor with a high mitotic rate that is characterized by ubiquitous pathogenic variants in *TP53* and alterations in *BRCA1* and *BRCA2*. In particular, 13–16% of these tumors present germline pathogenic variants of *BRCA1/2*, and 6% harbor somatic pathogenic variants [6,7,8]. Moreover, up to 51% of HGSCs have shown defects in the homologous recombination repair (HRR) pathway [8].

Deficiency in the HRR pathway leads cells to rely on more error-prone DNA repair systems [9], and therefore, over time, unrepaired double strand breaks (DSBs) induce the accumulation of genomic alterations such as insertions and deletions, copy number variations, and structural chromosomal rearrangements, leaving an irreversible “genomic scar” [10].

In HGSC, homologous recombination deficiency (HRD) is caused by pathogenic germline/somatic variants and epigenetic modifications in either *BRCA1/2* or in genes encoding for key actors in the HRR pathway. In fact, alterations in *RAD51B/C/D*, *PALB2*, *ATM*, *H2AX*, *CHK1/2*, *CDK12*, *NBN*, *MRE11*, *RPA*, *BRIP1*, *BARD1*, *RAD51*, Fanconi Anemia genes, *PTEN*, and *EMSY* have been shown to potentially confer an HRD or BRCAness phenotype, which is characterized by deficiencies in the DSB repair pathway [11,12].

HRD cells are particularly sensitive to poly (ADP-ribose) polymerase inhibitors (PARPis). In fact, inhibiting PARP1, which is crucial for single-strand DNA break repair, causes cell death in cells with impaired DSB repair capacity; this phenomenon is referred to as synthetic lethality [13].

The addition of PARPis to first-line chemotherapy regimens for women with platinum-sensitive ovarian cancer has improved clinical outcomes in terms of both progression-free and overall survival. Patients with pathogenic/likely pathogenic *BRCA* variants (*BRCAmut*) benefit the most from PARPis plus platinum treatment, but it is important to note that *BRCAwt*/HRD patients are also susceptible to such a therapy. In particular, the PRIMA trial showed median PFS for niraparib of 22.9, 19.6, and 8.1 months, respectively, in *BRCAmut*, *BRCAwt*/HRD and HR-proficient (HRp) patients; the PAOLA1 trial reported median PFS for olaparib of 37.2, 28.1, and 16.9 months, respectively, in *BRCAmut*, *BRCAwt*/HRD and HRp patients; and the VELIA trial showed median PFS for veliparib of 34.7, 22.9 and 15.0 months, respectively, in *BRCAmut*, *BRCAwt*/HRD, and HRp patients [14,15,16,17,18,19,20].

All these findings established the key role of both BRCA testing and HRD assessment in the treatment of HGSC patients. The American Society of Clinical Oncology (ASCO) recommends offering germline genetic testing for *BRCA1/2* to all women diagnosed with epithelial ovarian cancer, irrespective of their clinical features or family cancer history. Somatic tumor testing for *BRCA1/2* should be performed in women who do not carry a germline pathogenic or likely pathogenic *BRCA1/2* variant. Women with germline or somatic pathogenic or likely pathogenic *BRCA1/2* variants should be offered Food and Drug Administration (FDA)-approved treatments, such as PARPis [21].

Moreover, the FDA has recently approved HRD assays able to detect the related “genomic scar”, in order to stratify *BRCAwt* patients and consequently predict responses to platinum-based chemotherapy and synthetic lethal agents such as PARPis, and prognosis [10]. The most diffused assay to determine HRD status is MyChoice CDx (Myriad Genetics, Salt Lake City, UT, USA), which calculates a Genomic Instability Score (GIS) based on the evaluation of loss of heterozygosity (LOH), telomeric allelic imbalance (TAI), and large-scale state transitions (LST); furthermore, *BRCA1/2* variants are analyzed. Tumors with GIS < 42 are HRp, and tumors with GIS ≥ 42 and/or pathogenic *BRCA1/2* variants are defined as HRD.

Given the importance of HRD testing for patient management and treatment decision making, we wanted to assess the feasibility of a locally performed, commercially available HRD assay and to compare our findings to those obtained using Myriad MyChoice CDx. In addition, to further deepen the mechanisms behind HRD, we studied the HRR pathway by using both a custom NGS panel that analyzed 21 HRR-related genes and FISH analysis to determine the copy numbers of *PTEN* and *EMSY*.

## 2. Materials and Methods

### 2.1. Study Cohort

We analyzed a cohort of 16 patients diagnosed with HGSC at San Raffaele Hospital (Milan, Italy), in 2021. Patients’ clinicopathologic features are summarized in Table 1. The median age at diagnosis was 57.5 years old; the range was between 36 and 69 years old; median progression free survival (PFS) was 6.5 months. For 13 patients, the Myriad MyChoice CDx report was available.

### 2.2. DNA Extraction

DNA from FFPE HGSC was extracted from both surgical specimens (8 patients) and tissue biopsies (8 patients), depending on material availability at the time of analysis. An expert pathologist reviewed each case and selected the most representative areas of the tumor with a percentage of tumor cells above 50%. DNA extraction was performed using thee Maxwell^®^ RSC DNA FFPE kit and Maxwell RSC Instrument (Promega, Milan, Italy) and quantified using the Qubit DNA HS Assay Kit on Qubit 3.0 Fluorometer (ThermoFisher Scientific, Waltham, MA, USA), as previously described [22].

### 2.3. HRD Assay

HRD status was evaluated using the CE-IVD AmoyDx HRD Focus Panel (Amoy Diagnostics, Xiamen, China), according to the manufacturer’s instructions, using 80–100 ng DNA. DNA libraries were quantified using the Qubit 3.0 Fluorometer (Thermo Fisher Scientific, Waltham, MA, USA), and DNA fragment quality control was performed using the 2100 Bioanalyzer System and DNA 1000 kit (Agilent Technologies, Santa Clara, CA, USA). Sequencing was performed using the NextSeq500 platform and Mid v2 flow cell (Illumina, San Diego, CA, USA). Raw data were analyzed using the AmoyDx NGS Data Analysis System-ANDAS Software to detect *BRCA1/2* variants and HRD status. Samples with GIS ≥ 50 were considered HRD. *BRCA1/2* variants were classified according to the ACMG/ENIGMA 5-class system [23,24], thereby giving an accurate description of variants’ clinical significance.

### 2.4. Evaluation of HRR Pathway Genes Using a Next-Generation Sequencing (NGS) Custom Panel

Somatic alterations involving key players in the HRR pathway were analyzed utilizing a custom NGS panel developed with Thermo Fisher Scientific, the BRCA-Expanded Panel, on the Ion Torrent S5 (ThermoFisher Scientific). The assay analyzes the full-length coding sequences of 21 genes (*ATM, BARD1, BRCA1, BRCA2, BRIP1, CDK12, CHEK1, CHEK2, FANCD2, MRE11, NBN, PALB2, PARP1, RAD50, RAD51, RAD51B, RAD51C, RAD51D, RAD52, RAD54L, TP53*), using an input of 10 ng of DNA. Targeted libraries were prepared using Ion Chef^TM^ Instrument (ThermoFisher Scientific) following the manufacturer’s instructions and were sequenced on the 530^TM^ Chip (ThermoFisher Scientific). Raw data analysis was performed using Torrent Suite v5.12, as previously described [25]. Variants were classified in accordance with the ACMG/ENIGMA criteria and ClinVar/OncoKB databases.

### 2.5. Germline BRCA Testing

The presence of germline variants was assessed on genomic DNA extracted from patients’ peripheral whole blood samples (Maxwell^®^ RSC Whole Blood, Promega, Milan, Italy) by Sanger direct sequencing. After amplification, PCR products of the genetic variants and surrounding regions were purified using Clean PCR (CleanNA-PH Waddinxveen, Netherlands) and sequenced in both directions using a Big Dye Terminator v.1.1 Cycle Sequencing Kit (Applied Biosystems, Foster City, CA, USA). Sequencing products were purified using a Big Dye X-Terminator Kit (Applied Biosystems) and ran on an ABI 3730 Genetic Analyzer (Applied Biosystems). Called sequences were aligned to the reference using the Sequencer V.5.0 Software (Gene Codes Corporation, Ann Arbor, MI, USA) and classified in accordance with the ACMG/ENIGMA criteria [23,24].

### 2.6. FISH Analysis

*PTEN* homozygous deletion and *EMSY* copy number were assessed in all the study cases on 4 μm sections of FFPE tissue by fluorescence in situ hybridization (FISH) as previously described [24], using ZytoLight SPEC *PTEN*/CEN10 Dual Color Probe (ZytoVysion GmbH, Bremerhaven, Germany) and *EMSY* FISH Probe (Empire Genomics, Buffalo, NY, USA). FISH was performed according to the probe manufacturers’ suggested protocols, slides were analyzed using a Nikon 90i fluorescence microscope (Nikon Instruments SpA, Italy), and images were captured by Genikon software (Nikon, Tokyo, Japan).

*PTEN* and CEN10 signals were counted in a minimum of 60 nuclei, and a sample was considered to have deleted *PTEN* when both copies of the gene were lost in more than 20% of tumor cells. *EMSY* copies were counted in at least 60 tumor cells, and a sample was considered to have an increased copy number when the *EMSY* copy number was >4 copies/cell.

### 2.7. Statistical Analyses

Positive Percent Agreement (PPA), Negative Percent Agreement (NPA), and Overall Percent Agreement (OPA) were calculated to evaluate the performance of the AmoyDx HRD Focus Panel compared with that of the Myriad MyChoice CDx.

## 3. Results

### 3.1. Comparison of AmoyDx HRD Focus Panel and Myriad MyChoiceCDx for Assessing HRD Status

A cohort of 16 HGSC patients (Table 1), who were diagnosed at San Raffaele Hospital in 2021, was selected to assess the HRD status utilizing the CE-IVD AmoyDx HRD Focus Panel. This panel evaluates *BRCA1/2* status and can detect the HRD genomic scar by analyzing LOH, TAI, and LST. The DNA library concentration ranged between 34.8 and 50.4 ng/μL. The median concentration was 42.2 ng/μL. Thus, all samples were above the manufacturer’s recommended concentration of 20 ng/μL. All the libraries were of outstanding quality. The main peak of the DNA fragment size was between 258 and 268 bp, and the median value was 261 bp (Figure S1). Moreover, we obtained excellent sequencing metrics for all samples (Table S1).

As shown in Table 2 (left panel), thirteen samples were classified as HRD (GIS ≥ 50), whereas three samples (#3, #7, and #13) were identified as HR-proficient (HRD-). Five (#4, #6, #9, #10, and #12) of the thirteen HRD tumors had pathogenic or likely pathogenic *BRCA1/2* variants, and one sample (#16) had a variant of uncertain significance (VUS).

In addition, 13 out of 16 patients had access to the MyChoice CDx assay, which allowed us to compare the results of the two tests (Table 2, central panel) and obtain complete HRD status concordance, with Positive Percent Agreement (PPA), Negative Percent Agreement (NPA), and Overall Percent Agreement (OPA) values of 100%. The only discordant finding was patient #12: as a result of the existence of a likely pathogenic *BRCA2* variant, she was classified as HRD by both assays; nevertheless, the GIS was over the positivity threshold by the Amoy panel and below the positivity threshold by the Myriad panel.

The *BRCA1/2* variants identified by the two assays were identical in all cases. The five patients with pathogenic/likely pathogenic *BRCA1/2* variants identified on tumor samples were addressed for genetic counseling and germline genetic testing. Peripheral blood analysis revealed that *BRCA* variants in patients #4, #6, and #10 were germline (Table 2, right panel).

### 3.2. Investigation of HRR Pathway Gene Alterations

**Figure 1:**
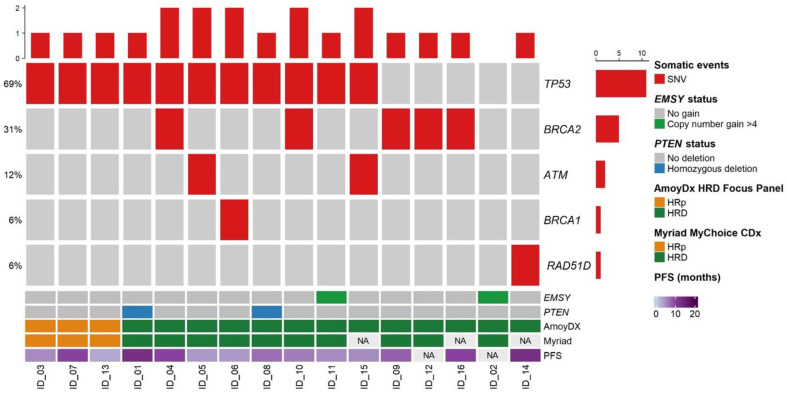
Oncoprint of mutated HRR genes identified in the study cohort. Cases sorted by HRD status. SNV were evaluated by BRCA-Expanded Panel, *PTEN* homozygous deletion, and *EMSY* copy number gain were evaluated by FISH analysis. NA = not available.

In order to better characterize the HRD phenotype, we analyzed genes encoding for key players in HRR by an NGS custom panel (BRCA-Expanded Panel). Pathogenic/likely pathogenic/uncertain variants are listed in Table 3; no benign/likely benign variants are reported. HR-proficient samples (patients #3, #7, and #13) were wild type for all the analyzed genes, except for *TP53* (Figure 1). Among the thirteen HRD patientst, 9 (patients #4, #5, #6, #9, #10, #12, #14, #15, and #16) had mutations in key HRR genes (*TP53*, *BRCA1*, *BRCA2*, *ATM*, and *RAD51D*), 3 had only *TP53* pathogenic variants (patients #1, #8 and #11), and 1 patient (#2) did not have any variant (Figure 1).

**Figure 2:**
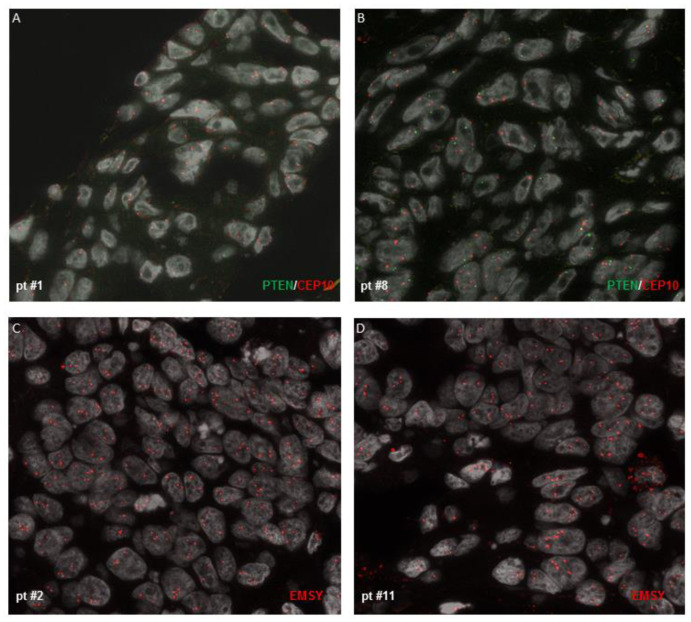
FISH analyses for *PTEN* and *EMSY*. *PTEN* homozygous deletion was observed in patient #1 (**A**) and in patient #8 (**B**). An elevated *EMSY* copy number was observed in patient #2 (**C**) and in patient #11 (**D**).

To further deepen the understanding of the mechanisms behind HRD, we investigated *PTEN* deletion and *EMSY* copy number by FISH analysis (Figure 1). We found homozygous deletion of *PTEN* in patients #1 and #8 (Figure 2A,B) and elevated *EMSY* copy numbers in patients #2 and #11 (Figure 2C,D). In all the other samples, we observed neither *PTEN* homozygous deletion nor *EMSY* copy number gain.

## 4. Discussion

Assessment of HRD status is now essential for ovarian cancer patient management. In fact, up to 51% of HGSCs have shown defects in the HRR pathway [8], and recent trials have demonstrated that not only patients with pathogenic/likely pathogenic *BRCA* variants, but also *BRCAwt*/HRD patients, are sensitive to PARPis and platinum therapy. PARPis have been recently approved by international medicine agencies for the treatment of ovarian cancer patients with either *BRCA* pathogenic variants or HRD, changing the ovarian cancer treatment landscape in both the first-line and relapsed disease settings [14,15,16,17,18,19,20]. The most common HRD test is Myriad MyChoice CDx, but there is a pressing need to offer an alternative to outsourcing analysis [26], which typically requires high costs and lengthy turnaround times.

In order to set up a complete in-house workflow for HRD testing, we analyzed a cohort of 16 HGSC patients using the CE-IVD AmoyDx HRD Focus Panel. This panel perfectly matches the requirements of a molecular diagnostic laboratory because it is simple to use, allows a 50% cost reduction when compared to the Myriad MyChoice CDx, and enables results with a turnaround time of 5 working days. It is important to emphasize that proper tumor sampling and fixation [27] allowed us (i) to recover DNA with sufficient quality and quantity for the assay from both surgical samples and biopsies, (ii) to obtain excellent sequencing metrics, and (iii) to successfully analyze all samples. Thirteen patients showed HRD, and three samples were HR-proficient. We found complete concordance in HRD status detected by the Myriad assay, which was available for 13 out of 16 patients. We are aware of the limited number of patients included in the study. This investigation was intended to be a proof of concept to determine both the feasibility and the performance of local HRD testing. Despite the small cohort, our results are extremely encouraging and in line with previous studies [28,29], highlighting the utility of such an approach and allowing us to include this test in the clinical molecular diagnostics routine.

The two assays identified the same variants in all patients with *BRCA1/2* alterations, but while the Myriad assay does not classify *BRCA* variants, grouping both pathogenic and likely pathogenic variants together as deleterious, the AmoyDx HRD assay adheres to the ACMG/ENIGMA 5-class system, giving a more accurate description of variants’ clinical significance [23].

It is important to underline that one of the limitations of HRD tests is the absence of information on the HRR-related genes beyond *BRCA1/2*. Thus, we analyzed 21 genes involved in the HRR pathway by using the BRCA-Expanded Panel. We detected the same pathogenic/likely pathogenic *BRCA1/2* variants as in the HRD tests, but we also found SNV variants in *ATM* (patient #5 and patient #15), and in *RAD51D* (patient #14). Further, we investigated the homozygous deletion of *PTEN* and copy number gain of *EMSY* by FISH analysis. In fact, PTEN deficiency leads to impaired RAD51-mediated DSB repair and genomic instability, thereby causing HR deficiency and leading to sensitivity to PARPis, both in vitro and in vivo [30,31]. The copy number gain of *EMSY*, which binds to *BRCA2*, exon 3, has been described in 17% of high-grade ovarian cancer, and it has been reported as an alternative mechanism for HRD, even if its role is controversial and it does not seem to confer sensitivity to PARPis [32,33,34]. We found that patients #1 and #8 had homozygous deletion of *PTEN*, and patients #2 and #11 had elevated *EMSY* copy numbers, suggesting that these alterations may have been the causes of HRD in the four patients. Additionally, the presence of either *PTEN* homozygous deletion or increased *EMSY* copy number was not concurrent with alterations in other HRR key players. However, due to the small size of the study group, a larger cohort should be used to further corroborate this observation.

Among the nine HRD patients treated with PARPis, three (33%) experienced tumor progression, and six (67%) do not show evidence of disease. PARPi resistance has already been reported in the literature; therefore, for patient #6, carrying a *BRCA1* pathogenic variant, we could hypothesize several causing events, such as the occurring of alterations in *PARP1*, the loss of *PARG*, and reversion mutations in *BRCA1* or other HRR genes; moreover, loss of *TP53BP1, RIF1, REV7*, or *DYNLL1* has been associated with PARPi resistance in BRCA1-deficient cells [35,36]. For patients #5 and #11, as the *ATM* variant is of uncertain significance (VUS) (patient #5) and the role of *EMSY* gene copy number increase (patient #11) is still under investigation, further studies are required to establish their significance in drug responses.

The patients with no evidence of disease after at least 6 months were characterized by *PTEN* homozygous deletion (#1 and #8), *BRCA2* pathogenic variants (#4, #9, and #10), and *RAD51D* VUS (#14). This last patient experienced recurrence after chemotherapy and is now showing sensitivity to niraparib. Interestingly, such a finding could support the in silico prediction of a deleterious effect of this variant on protein structure/function, in line with the association between *RAD51D* loss-of-function variants and sensitivity to PARPis [37,38].

In conclusion, this is one of the first studies to compare the Myriad assay with the results of a locally performed test, showing excellent concordance and short turnaround times. Despite the restriction of a small cohort of samples, our findings support the feasibility of internal HRD testing, which has now become essential for the management of ovarian cancer. Importantly, this strategy could be also applied in the near future to stratify patients with different tumor types [39], including breast cancer, pancreatic cancer, and prostate cancer, thereby expanding the number of patients who could benefit from PARPi treatment.

## 5. Conclusions

Updated ovarian cancer patient therapy selection requires assessment of the HRD status; therefore, it is advantageous that such analysis is locally available, avoiding the higher costs and lengthy turnaround times of outsourcing. Our data demonstrated the feasibility of internal HRD testing for HGSC and designed an HRD testing strategy that may be extended to other tumor types that could benefit from PARPi treatment.

## Figures and Tables

**Table 1:** Patients’ clinicopathological features.

Pt	Age	Sample	pT	G	pN	Therapy	Maintenance	PFS	Progr	Recur	Currently
ID_01	47	SS	3c	IV	1a	C + T + B	B + Ola	13			NED-maintenance ongoing
ID_02	50	SS	3c	IV	x	C + T + B	B	FU lost °			/
ID_03	68	SS	3b	IV	x	C + T	NO	5		yes	under evaluation
ID_04	59	SS	3c	IV	0	C + T	Ola	10			NED-maintenance ongoing
ID_05	36	Bx		III		C + T + IDS	Nira	4	yes		T + B ongoing
ID_06	43	Bx		IV		C + T + IDS	Ola	4	yes		T + B ongoing
ID_07	61	Bx		III		C + T + IDS + B	B	10			NED-B ongoing
ID_08	39	Bx		III		C + T + IDS	Nira *	7			NED
ID_09	51	SS	3c	III/IV	1a	C + T	Ola	8			NED-Ola ongoing
ID_10	56	Bx		III		C + T + IDS	Ola	6			NED-Ola ongoing
ID_11	66	Bx		-		C + T	Nira	5	yes		
ID_12	69	Bx		IV		C + T + IDS					starting Ola
ID_13	67	SS	3c	IV	0	C + T	Nira	3			NED-Nira ongoing
ID_14	53	Bx		III		C + T	NO ^	13		yes	C + T; Nira ongoing
ID_15	68	SS	2b	III	x	C + T	NO ^	5			NED
ID_16	62	SS	2b	III	0	C + T	NO ^	10			NED

SS = surgical specimen; Bx = biopsy; G = grade; pT = tumor stage; pN = lymph node stage (according to WHO/TNM); C = carboplatin; T = taxol; B = Bevacizumab; IDS = interval debulking surgery; Ola = Olaparib; Nira = niraparib; PFS = progression-free survival, in months; FU = follow up; Progr = progression; Recur = recurrence; NED = no evidence of disease; ° patient moved to another hospital; * interrupted because of neutropenia; ^ no maintenance therapy because of tumor stage.

**Table 2:** Results of AmoyDx HRD Focus Panel (left), Myriad MyChioce CDx (central), and BRCA somatic and germline tests (right).

	AmoyDx HRD Focus Panel				Myriad MyChoice CDx				*BRCA1/2* Testing	
Pt	HRD	GIS	*BRCA*	Significance	HRD	GIS	*BRCA*	Significance	Somatic ^$^	Germline
ID_01	+	99.2	-	All benign	+	72	-	All benign	WT	
ID_02	+	100	-	All benign	+	51	-	All benign	WT	
ID_03	-	45.4	-	All benign	-	28	-	All benign	WT	
ID_04	+	98.3	+	Pathogenic	+	60	+	Deleterious	* *BRCA2* p.Tyr1739Ter (c.5217_5220del) 89%	yes
ID_05	+	98.3	-	All benign	+	66	-	All benign	WT	
ID_06	+	97.3	+	Pathogenic	+	54	+	Deleterious	* *BRCA1* p.Tyr777Ter (c.2331T > G) 76%	yes
ID_07	-	36.1	-	Likely-benign	-	36	-	All benign	WT	
ID_08	+	97.1	-	All benign	+	48	-	All benign	WT	
ID_09	+	97.6	+	Likely-pathogenic	+	60	+	Deleterious	* *BRCA2* p.Ala1327ProfsTer8 (c.3979delG) 76%	no
ID_10	+	96.8	+	Pathogenic	+	57	+	Deleterious	* *BRCA2* p.Val1283LysfsTer2 (c.3847_3848delGT) 79%	yes
ID_11	+	100	-	All benign	+	67	-	All benign	WT	
ID_12	+	59.1	+	Likely-pathogenic	+	36	+	Deleterious	* *BRCA2* p.Asn615ThrfsTer29 (c.1842delT) 24%	no
ID_13	-	14.1	-	All benign	-	22	-	All benign	WT	
ID_14	+	98.9	-	All benign	na	na			WT	
ID_15	+	72	-	All benign	na	na			WT	
ID_16	+	84	+	Uncertain	na	na			^ *BRCA2* p.Arg2991Cys (c.8971C > T) 31%	

HRD+ = HRD; HRD- = HRp; na = not available; *BRCA1* (NM_007294.4), *BRCA2* (NM_000059); ^$^ evaluated using BRCA-Expanded Panel; * *BRCA* variants identified by AmoyDx HRD Focus Panel, Myriad MyChoice CDx, and BRCA-Expanded Panel; ^ *BRCA* variant identified by AmoyDx HRD Focus Panel and BRCA-Expanded Panel.

**Table 3:** Gene variants identified by BRCA-Expanded Panel.

Pt	HRD	BRCA-Expanded Panel		
		Variant	VAF	Significance
ID_01	+	*TP53* p.Pro190Thr (c.568C > A)	82%	VUS
ID_02	+	WT		
ID_03	-	*TP53* p.Arg175His (c.524G > A)	73%	Pathogenic
ID_04	+	*BRCA2* p.Tyr1739Ter (c.5217_5220del)	89%	Pathogenic
ID_05	+	*ATM* p.His2552Asn (c.7654C > A)	44%	VUS
ID_06	+	*BRCA1* p.Tyr777Ter (c.2331T > G)	76%	Pathogenic
ID_07	-	*TP53* p.Arg175His (c.524G > A)	64%	Pathogenic
ID_08	+	*TP53* p.Cys141Tyr (c.422G > A)	90%	Pathogenic
ID_09	+	*BRCA2* p.Ala1327ProfsTer8 (c.3979delG)	76%	Likely-pathogenic
ID_10	+	*BRCA2* p.Val1283LysfsTer2 (c.3847_3848delGT)	79%	Pathogenic
ID_11	+	*TP53* p.Tyr163Cys (c.488A > G)	48%	Pathogenic
ID_12	+	*BRCA2* p.Asn615ThrfsTer29 (c.1842delT)	24%	Likely-pathogenic
ID_13	-	*TP53* p.Cys182AlafsTer65 (c.544delT)	72%	Likely-pathogenic
ID_14	+	*RAD51D* p.Cys9Ser (c.26G > C)	71%	VUS
ID_15	+	*ATM* p.Tyr454His (c.1360T > C)	40%	VUS
ID_16	+	*BRCA2* p.Arg2991Cys (c.8971C > T)	31%	VUS

HRD+ = HRD; HRD- = HRp VAF = variant allele frequency; VUS = variant of uncertain significance; *TP53* (NM_000546.5); *BRCA1* (NM_007294.4); *BRCA2* (NM_000059); *ATM* (NM_000051.3); *RAD51D* (NM_133629.2).

## Data Availability

The data presented in this study are available on request from the corresponding author.

## Supplementary Materials

The following supporting information can be downloaded at: https://www.mdpi.com/article/10.3390/cancers15010043/s1. Figure S1: Bioanalyzer densitometry plots of DNA libraries. Gel-like images show the expected main peak of 258–268 bp for all samples. Table S1: Sample sequencing metrics of AmoyDx HRD Focus Panel.
