# Supplementary material for: Locally Performed HRD Testing for Ovarian Cancer? Yes, We Can!
Source: Cancers (Basel). 2022 Dec 21;15(1):43. doi: 10.3390/cancers15010043 (PMC9817883; doi:10.3390/cancers15010043)
Supplement: Supplementary file 1 [file cancers-15-00043-s001.zip › Figure S1.pptx]

## Slide 1
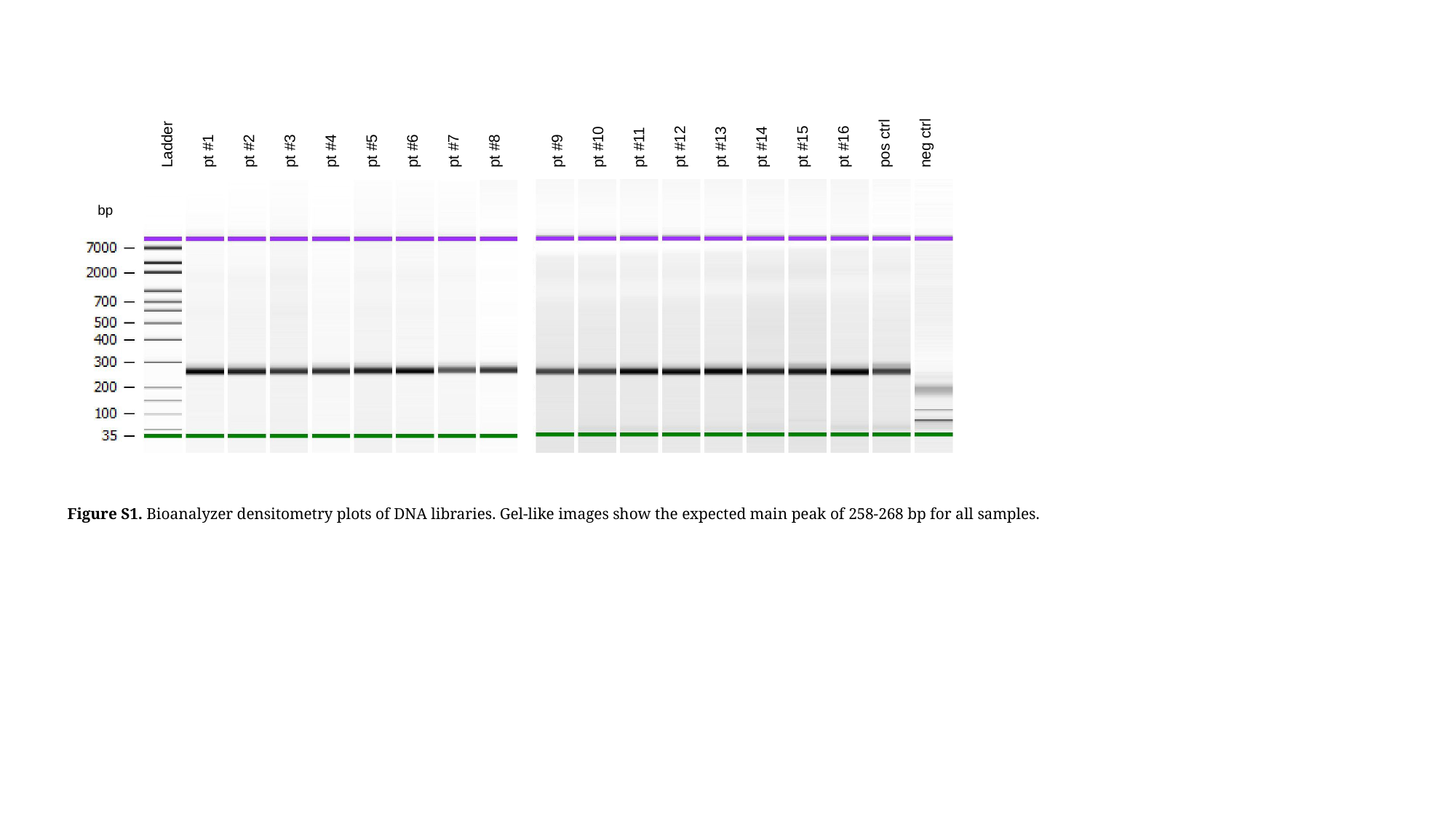

Ladder
pt #1
pt #2
pt #3
pt #4
pt #5
pt #6
pt #7
pt #8
pt #9
pt #10
pt #11
pt #12
pt #13
pt #14
pt #15
pt #16
pos ctrl
neg ctrl
 bp
Figure S1. Bioanalyzer densitometry plots of DNA libraries. Gel-like images show the expected main peak of 258-268 bp for all samples.
