# Supplementary material for: Locally Performed HRD Testing for Ovarian Cancer? Yes, We Can!
Source: Cancers (Basel). 2022 Dec 21;15(1):43. doi: 10.3390/cancers15010043 (PMC9817883; doi:10.3390/cancers15010043)
Supplement: Supplementary file 1 [file cancers-15-00043-s001.zip › Table S1.pdf]

Table S1. Sample sequencing metrics of AmoyDx HRD Focus Panel

Pt	Total Reads	Q20	Q30	Alignment	Clean Match	Coverage	BRCA EffectiveDepth	BRCA Uniformity	BRCA CV	SNP EffectiveDepth	SNP Uniformity	SNP CV	GIS
ID_01	23,799,410	98.61%	97.88%	98.96%	98.11%	99.92%	1107	100.00%	0.32	278	96.71%	0.62	99.2
ID_02	26,361,786	98.56%	97.81%	99.06%	98.17%	99.94%	1898	99.61%	0.33	342	95.89%	0.60	100
ID_03	32,662,858	97.81%	96.62%	98.94%	98.18%	99.93%	3010	100.00%	0.32	446	95.65%	0.69	45.4
ID_04	26,723,692	98.63%	97.89%	99.22%	98.27%	99.94%	1247	99.61%	0.40	366	96.36%	0.70	98.3
ID_05	27,304,074	98.53%	97.75%	99.02%	98.05%	99.92%	2729	100.00%	0.25	351	95.93%	0.56	98.3
ID_06	29,114,910	98.05%	96.97%	99.11%	98.20%	99.92%	3393	100.00%	0.27	453	96.95%	0.61	97.3
ID_07	26,925,444	98.52%	97.74%	99.07%	98.19%	99.94%	3942	99.61%	0.29	411	96.10%	0.60	36.1
ID_08	26,871,078	98.57%	97.83%	99.06%	98.12%	99.94%	1670	99.61%	0.27	319	95.36%	0.65	97.1
ID_09	33,853,096	97.98%	96.87%	99.07%	98.23%	99.92%	2439	99.61%	0.25	540	96.68%	0.66	97.6
ID_10	34,407,574	97.80%	96.60%	98.58%	97.58%	99.92%	2454	99.61%	0.33	324	95.73%	0.60	96.8
ID_11	30,663,268	97.93%	96.78%	98.95%	98.13%	99.92%	3177	100.00%	0.28	415	95.12%	0.62	100
ID_12	25,169,280	98.54%	97.78%	99.15%	98.30%	99.92%	1726	99.61%	0.34	325	97.25%	0.56	59.1
ID_13	27,032,192	98.49%	97.69%	99.27%	98.30%	99.95%	2034	100.00%	0.27	466	96.93%	0.66	14.1
ID_14	31,517,690	97.84%	96.67%	98.83%	97.84%	99.93%	2658	100.00%	0.28	368	97.02%	0.56	98.9
ID_15	31,566,644	97.90%	96.75%	98.85%	98.06%	99.92%	2104	100.00%	0.29	313	96.71%	0.60	72
ID_16	38,562,918	97.80%	96.61%	98.93%	98.10%	99.93%	3679	100.00%	0.21	593	96.50%	0.72	84

Optimum parameters as indicated by Amoy protocol: Q20 ≥ 85%; Q30 ≥ 75%; Alignment ≥ 85%; Clean Match ≥ 85%; Coverage ≥ 98%; BRCA Effective Depth ≥ 400x; BRCA Uniformity ≥ 95%; BRCA CV: the smaller the better; SNP Effective Depth ≥200x; SNP Uniformity ≥ 90%; SNP CV: the smaller the better
